# Ulinastatin inhibits cerebral ischemia-induced apoptosis in the hippocampus of gerbils

**DOI:** 10.3892/mmr.2015.3612

**Published:** 2015-04-15

**Authors:** YOUNG-SAM CHO, MAL-SOON SHIN, IL-GYU KO, SUNG-EUN KIM, CHANG-JU KIM, YUN-HEE SUNG, HYE-SUN YOON, BONG-JAE LEE

**Affiliations:** 1 Department of Urology, Kangbuk Samsung Hospital, Sungkyunkwan University School of Medicine, Seoul 110-746, Republic of Korea; 2 Department of Physiology, Kyung Hee University College of Medicine, Seoul 130-701, Republic of Korea; 3 Department of Physical Therapy, Kyungnam University, Changwon 631-701, Republic of Korea; 4 Department of Pediatrics, Eulji General Hospital, Eulji University School of Medicine, Seoul 139-872, Republic of Korea; 5 Department of Anesthesiology and Pain Medicine, Kang Dong Kyung Hee Hospital, Kyung Hee University College of Medicine, Seoul 134-727, Republic of Korea

**Keywords:** ulinastatin, transient global ischemia, apoptotic cell death, cell proliferation, hippocampus, short-term memory

## Abstract

Ulinastatin is a urinary trypsin inhibitor, originally extracted and purified from human urine. Ulinastatin has cytoprotective effects against ischemic injury in several organs. In the present study, the neuroprotective effects of ulinastatin following ischemic cerebral injury in the hippocampus of gerbils was investigated. To induce transient global ischemia in gerbils, the common carotid arteries were occluded using aneurysm clips for 5 min, and the clips were then removed. Ulinastatin was subcutaneously injected into the gerbils once a day for 7 days at doses of 50,000 or 100,000 U/kg. The gerbils were confronted with a step-down avoidance task, following which tissue samples from the gerbils were examined using terminal deoxynucleotidyl transferase-mediated dUTP nick end labeling (TUNEL) staining, western blot analysis for B-cell lymphoma (Bcl-2) and Bcl-2-associated X protein (Bax), immunohistochemistry for caspase-3 and immunofluorescence for 5-bromo-2′-deoxyuridine. The numbers of TUNEL-positive and caspase-3-positive cells in the hippocampal CA1 region increased following cerebral ischemia. The expression of Bax in the hippocampus increased, while the expression of Bcl-2 in the hippocampus decreased following cerebral ischemia. These results confirmed that apoptosis in the hippocampus was enhanced following cerebral ischemia in gerbils. The levels of cell proliferation in the hippocampal dentate gyrus were also enhanced by ischemia, which is possibly an adaptive mechanism to compensate for excessive levels of apoptosis. Ulinastatin treatment inhibited ischemia-induced apoptosis by suppressing apoptosis-associated molecules, and thus ameliorated ischemia-induced short-term memory impairment. The cell proliferation in the hippocampus was also suppressed following ulinastatin treatment. These results suggested the use of ulinastatin as a therapeutic agent for patients with cerebral stroke.

## Introduction

Ischemic injury in the brain leads to neuronal cell death, and neuronal cell death following cerebral ischemia is caused by the interaction of complex processes, including excitotoxicity, depolarization, inflammation and apoptosis (1,2). Apoptosis is one of the major pathways leading to cell death in cerebral ischemia injury (3). Cerebral ischemia-induced apoptosis in the hippocampus causes memory impairment (4–6).

Terminal deoxynucleotidyl transferase-mediated dUTP nick end labeling (TUNEL) staining detects DNA fragmentation, which is one of the hallmarks of apoptosis. Two important groups of proteins in the apoptotic cascades are members of the B-cell lymphoma (Bcl)-2 family and classes of caspases (7). The Bcl-2 family can be classified into two functionally distinct groups: Anti-apoptotic proteins and pro-apoptotic proteins. Bcl-2, an anti-apoptotic protein, is known to regulate apoptotic pathways and protect against cell death. Bcl-2-associated X protein (Bax), a pro-apoptotic protein of the Bcl-2 family, is expressed in high levels and selectively during apoptosis, and promotes cell death (7–9). The increment of the ratio of Bax to Bcl-2 represents the induction of apoptosis in several tissues (8,9). Caspase-3 is one of the key executors of apoptosis, and activation of caspase-3 is implicated in apoptotic neuronal cell death in animal models of stroke (1,10).

The adult central nervous system contains stem cells, which are capable of generating new neurons. It has been suggested that increased neurogenesis in the hippocampal dentate gyrus may promote morphological and functional recovery (11,12). 5-Bromo-2′-deoxyuridine (BrdU) is an indicator of DNA synthesis. It is incorporated into newly synthesized DNA during the S-phase of the cell cycle, prior to cell division, and during the repair of damaged DNA (13). The number of BrdU-positive cells increases following cerebral ischemia, and this increase in proliferation following brain damage, including during stroke, has been suggested as an adaptive mechanism to compensate for excessive apoptosis (14,15).

Ulinastatin is a urinary trypsin inhibitor, originally extracted and purified from human urine, and is an intrinsic serine-protease inhibitor (16). Urinary trypsin inhibitors have anti-inflammatory activities, which suppress the infiltration of neutrophils, and their release of elastase and chemical mediators (17,18). Although the cytoprotective effects of ulinastatin in ischemic injury have been observed in several organs (19,20), the neuroprotective effects of ulinastatin in ischemic cerebral injury, and their association with levels of apoptotic molecules remain to be eluicidated.

In the present study, the effects of ulinastatin on short-term memory, apoptotic neuronal cell death and cell proliferation in the hippocampal regions were investigated following transient global ischemia in gerbils. The gerbils were provided with a step-down avoidance task, and tissue samples were then obtained and analyzed using TUNEL staining, western blot analysis for Bax and Bcl-2, immunohistochemistry for caspase-3 and immunofluorescence for BrdU.

## Materials and methods

### Experimental animals

Adult male Mongolian gerbils (11–13-week-old) were used in the present study. The experimental procedures were performed in accordance with the animal care guidelines of the National Institutes of Health (NIH) and the Korean Academy of Medical Sciences (Seoul, Korea), and the study was approved by the Kyung Hee University Institutional Animal Care and Use Committee (Seoul, Korea). The gerbils were housed under controlled temperature (20±2°C) and lighting (07:00 to 19:00 h) conditions, with food and water made available *ad libitum*. The gerbils were randomly divided into four groups (n=10 in each group): The sham group, the ischemia group, the ischemia and 50,000 units ulinastatin group, and the ischemia and 100,000 units ulinastatin group.

Beginning 1 day after the induction of ischemia, ulinastatin (Wakamoto Pharmaceutical Co., Tokyo, Japan) was subcutaneously injected into the gerbils in the ulinastatin-treated groups once a day for 7 consecutive days at a dose of either 50,000 or 100,000 U/kg. The gerbils in the sham and ischemia group were administered with saline subcutaneously once a day for 7 days. BrdU (50 mg/kg; Sigma-Aldrich, St. Louis, MO, USA) was administered intraperitoneally to all the animals 1 h prior to ulinastatin treatment, once each day for the same duration.

### Induction of transient global ischemia

To induce transient global ischemia in the gerbils, a surgical procedure was performed, according to a previously described experimental method (5). In brief, the gerbils were anesthetized with 3% isoflurane (JW Pharmaceutical Corporation, Seoul, Korea) in 20% O_2_-77% N_2_. Following bilateral neck incisions, the common carotid arteries were exposed and occluded with aneurysm clips (Fine Science Tools, Foster City, CA, USA) for 5 min. The clips were then removed to restore cerebral blood flow. Body temperatures were maintained at 37±0.5°C, measured rectally, with a heating lamp (Harvard Apparatus, Holliston, MA, USA) until the gerbils regained consciousness. Following recovery, the animals were monitored for an additional 2 h to prevent hypothermia. The gerbils in the sham group underwent the same surgical procedures, with the exception of the common carotid artery occlusion.

### Step-down avoidance task

Latency in a step-down avoidance task was measured to evaluate short-term memory, as previously described (21). The gerbils were trained in the step-down avoidance task 7 days from the start of ulinastatin treatment. The gerbil was placed on a 7×25 cm platform, which was 2.5 cm in height. The platform faced a 45×25 cm grid of parallel stainless steel bars, 0.1 cm in caliber, spaced 1 cm apart. In the training session, the animal received a 0.2 mA scramble foot shock for 2 sec immediately upon stepping down. At 2 h after the training session, the latency (sec) in each group was measured. The time elapsed between when the gerbil was placed on the platform and when the gerbil stepped down and placed all four paws on the grid was defined as the latency. A latency >180 sec was counted as 180 sec.

### Tissue preparation

The gerbils were sacrificed immediately following the determinination of latency. The animals were anesthetized using Zoletil 50^®^ (10 mg/kg intraperitoneally; Vibac Laboratories, Carros, France), transcardially perfused with 50 mM phosphate-buffered saline (PBS), and fixed with a freshly prepared solution consisting of 4% paraformaldehyde (Junsei Chemical Co., Ltd., Tokyo, Japan) in 100 mM phosphate buffer (pH 7.4; Thermo Fisher Scientific, Waltham, MA, USA). The brains were dissected and post-fixed in the same fixative overnight, and then transferred into a 30% sucrose solution (Sigma-Aldrich) for cryoprotection. Coronal sections of 40 *µ*m thickness were made using a freezing microtome (CM 1510-3; Leica, Nussloch, Germany).

### TUNEL staining

For visualization of DNA fragmentation, a marker of apoptotic cell death, TUNEL staining was performed using an In Situ Cell Death Detection kit^®^ (Roche, Mannheim, Germany), according to the manufacturer’s instructions (5). To begin the procedure, 10 sections were post-fixed in ethanol-acetic acid (2:1; 500 *µ*l/section; Sigma-Aldrich) at −20°C, and rinsed with PBS. The sections were then incubated with proteinase K (100 *µ*g/ml; 5 *µ*l/section; Sigma-Aldrich), rinsed, incubated with 3% H_2_O_2_ (500 *µ*l/section; Sigma-Aldrich) at room temperature, permeabilized with 0.5% Triton X-100 (500 *µ*l/section; Sigma-Aldrich) at −20°C, rinsed again, and then incubated in the TUNEL reaction mixture (6 *µ*l/section) at 37°C. The sections were then rinsed and were visualized using Converter-POD with 0.02% 3,3′-diaminobenzidine (DAB; 100 *µ*l/section; Sigma-Aldrich) at room temperature. Mayer’s hematoxylin (100 *µ*l/section; DAKO, Glostrup, Denmark) was used for counter-staining at room temperature, and the sections were finally mounted onto gelatin-coated slides (Marienfeld-Superior, Lauda-Königshofen, Germany). The slides were air dried overnight at room temperature, and coverslips were mounted using Permount^®^ (Thermo Fisher Scientific).

### Caspase-3 immunohistochemistry

For visualization of the expression of caspase-3, caspase-3 immunohistochemistry was performed, according to the method described previously (5). A total of 10 sections were removed from each brain and incubated overnight at 4°C with mouse polyclonal anti-caspase-3 antibody (1:500; cat. no. sc-7272; Santa Cruz Biotechnology, Inc., Santa Cruz, CA, USA) and washed three times with PBS. The sections were then incubated for another 1 h at room temperature with biotinylated horse anti-mouse secondary antibody (1:100; cat. no. BA2000; Vector Laboratories, Burlingame, CA, USA), and washed three times with PBS. The bound secondary antibody was then amplified using a Vector Elite ABC kit® (Vector Laboratories). The antibody-biotin-avidin-peroxidase complexes were visualized using 0.02% DAB, and the sections were finally mounted onto gelatin-coated slides. The slides were air dried overnight at room temperature, and coverslips were mounted using Permount^®^ (Thermo Fisher Scientific).

### BrdU immunofluorescence

For the detection of newly-generated cells in the dentate gyrus, BrdU-specific immunofluorescence was performed, as previously described (5). The sections were first permeabilized by incubation with 0.5% Triton X-100 in PBS (1 ml/well) for 20 min at room temperature, and were then pretreated with 50% formamide-2X standard saline citrate (1 ml/well) at 65°C for 2 h, denatured in 2N HCl (2 ml/well) at 37°C for 30 min, and rinsed twice in 100 mM sodium borate (pH 8.5; 1 ml/well). For double labeling of BrdU and neuronal nuclear antigen (NeuN), the sections were incubated overnight with rat anti-BrdU antibody (1:300, Abcam, Biomeda, CA, USA) and mouse anti-NeuN antibody (1:500, Chemicon International, Temecula, CA, USA) at 4°C overnight following DNA denaturation. The sections were then incubated for 2 h with cy3-conjugated anti-rat secondary antibody for BrdU (1:200, Jackson ImmunoResearch Laboratories, West Grove, PA, USA) and fluorescein isothiocyanate-conjugated anti-mouse secondary antibody for NeuN (1:200; Jackson ImmunoResearch Laboratories). The sections were then mounted on gelatin-coated glass slides, and the coverslips were mounted using fluorescent mounting medium (DakoCytomation, Carpinteria, CA, USA). Images of the fluorescent staining were captured using an epifluorescent microscope (Nikon Eclipse 50i; Nikon Inc., Melville, NY, USA).

### Western blot analysis

Western blot analysis was performed to detect the protein expression levels of Bax and Bcl-2 in the hippocampus, as previously described (10). The hippocampal tissues were collected and immediately frozen at −70°C. The hippocampal tissue samples were homogenized on ice, and lysed in lysis buffer containing 50 mM HEPES (pH 7.5), 150 mM NaCl, 10% glycerol, 1% Triton X-100, 1 mM phenylmethylsulfonyl fluoride, 1 mM ethylene glycol tetraacetic acid, 1.5 mM MgCl_2_·6H_2_O, 1 mM sodium orthovanadate, and 100 mM sodium fluoride (Sigma-Aldrich). The protein content was measured using a DC Universal Protein Assay kit (Bio-Rad Laboratories, Inc., Hercules, CA, USA). Equal quantities of total protein (30 *µ*g) from each group were separated on 12% SDS-polyacrylamide gels and transferred onto a nitrocellulose membrane (GE Healthcare Life Sciences, Piscataway, NJ, USA). Mouse actin antibody (1:500; Santa Cruz Biotechnology, Inc.), mouse Bax antibody (cat. no. SC7480; 1:1000; Santa Cruz Biotechnology, Inc.), and mouse Bcl-2 antibody (cat. no. SC7382; 1:1000; Santa Cruz Biotechnology, Inc.) were used as the primary antibodies and incubated overnight at 4°C. Horseradish peroxidase-conjugated anti-mouse antibody for Bax and Bcl-2 (cat. no. RPN4201; 1:2,000; Amersham Pharmacia Biotechnology GmbH, Freiburg, Germany) were used as the secondary antibodies and incubated for 1 h at room temperature. Experiments were performed under normal laboratory conditions and at room temperature, with the exception of the treatment of the transfer membranes. The transfer membranes were treated at 4°C with a cold pack and pre-chilled transfer buffer (25 mM Tris, 192 mM glycine, pH 8.3). Band detection was performed using an enhanced chemiluminescence (ECL) detection kit (cat. no. SC2048; Western Blotting Luminol Reagent; Santa Cruz Biotechnology, Inc.), at a wavelength of 428 nm.

### Data analysis

The numbers of TUNEL-positive and caspase-3-positive cells in the hippocampal CA1 region, and the number of BrdU-positive cells in the hippocampal dentate gyrus were counted using an Image-Pro^®^ Plus computer-assisted image analysis system (Media Cyberbetics Inc., Silver Spring, MD, USA) attached to a light microscope (BX-51; Olympus Corporation, Tokyo, Japan) under x10 magnification. To compare the relative expression of proteins, the band intensities were quantified densitometrically using Molecular Analyst™ version 1.4.1 (Bio-Rad Laboratories, Inc.).

Statistical analysis was performed using one-way analysis of variance followed by Duncanm’s post-hoc test. Statistical analyses were conducted using SPSS version 21.0 (IBM, Armonk, NY, USA). The results are expressed as the mean ± standard error of the mean. P<0.05 was considered to indicate a statistically significant difference.

## Results

### Effect of ulinastatin on latency in the step-down avoidance task

**Figure 1:**
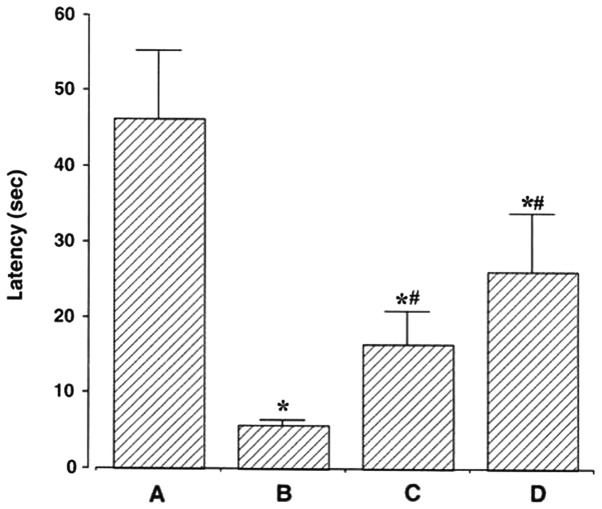
Effect of ulinastatin on latency in a step-down avoidance task. (A) Sham-operation, (B) ischemia-induction, (C) ischemia-induction and 50,000 units ulinastatin, (D) ischemia-induction and 100,000 units ulinastatin. Data are presented as the mean ± standard error of the mean. *P<0.05, compared with the sham-operation group; ^#^P<0.05, compared with the ischemia-induction group.

The latencies observed in the step-down avoidance task are presented in Fig. 1. Latency was 46.38±9.04 sec in the sham group, 5.67±0.82 sec in the ischemia group, 16.60±4.27 sec in the ischemia and 50,000 units ulinastatin group, and 26.00±7.78 sec in the ischemia and 100,000 units ulinastatin group. These results demonstrated that ischemic insult caused deterioration of short-term memory, and that ulinastatin alleviated the ischemia-induced impairment in short-term memory.

### Effect of ulinastatin on the numbers of TUNEL-positive and caspase-3-positive cells in the hippocampal CA1 region

**Figure 2:**
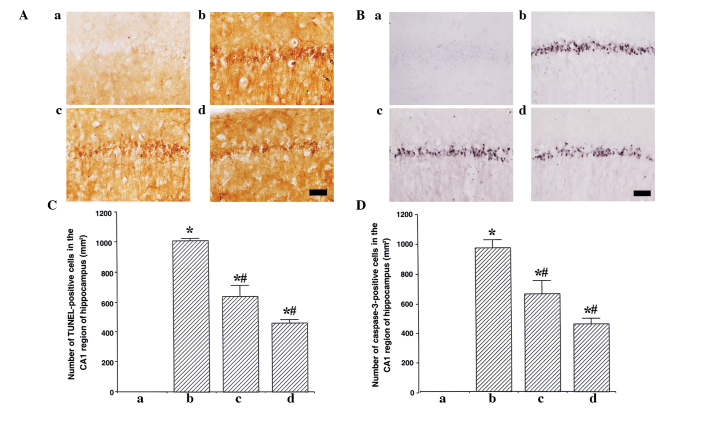
Effects of ulinastatin on DNA fragmentation and the expression of caspase-3 in the hippocampal CA1 region. (A) Photomicrographs of terminal TUNEL-positive cells. (B) Number of TUNEL-positive cells. (C) Photomicrograph of caspase-3-positive cells. (D) Number of caspase-3-positive cells. All scale bars=50 *µ*m. (a) Sham-operation, (b) ischemia-induction, (c) ischemia and 50,000 units ulinastatin, (d) ischemia and 100,000 units ulinastatin. Data are presented as the mean ± standard error of the mean. *P<0.05, compared with the sham-operation group; ^#^P<0.05, compared with the ischemia-induction group. TUNEL, deoxynucleotidyl transferase-mediated dUTP nick end labeling.

Photomicrographs of TUNEL-positive cells in the hippocampal CA1 region are presented in Fig. 2A. The number of TUNEL-positive cells was 0.00±0.00/mm^2^ in the sham group, 1,007.19±41.32/mm^2^ in the ischemia group, 635.66±73.51/mm^2^ in the ischemia and 50,000 units ulinastatin group, 456.79±21.38/mm^2^ in the ischemia and 100,000 units ulinastatin group Fig. 2B). These results demonstrated that ischemic insult enhanced DNA fragmentation in the CA1 region, and that ulinastatin treatment suppressed the ischemia-induced DNA fragmentation.

Photomicrographs of caspase-3-positive cells in the hippocampal CA1 region are presented in Fig. 2C. The number of caspase-3-positive cells was 0.78±0.01/mm^2^ in the sham group, 964.80±52.31/mm^2^ in the ischemia group, 654.45±87.82/mm^2^ in the ischemia and 50,000 units ulinastatin group, and 447.74±38.42/mm^2^ in the ischemia-and 100,000 units ulinastatin group (Fig. 2D). These results demonstrate that ischemic insult enhanced the expression of caspase-3 in the CA1 region, and that ulinastatin treatment suppressed the ischemia-induced expression of caspase-3.

### Effect of ulinastatin treatment on the protein expression of Bcl-2 and Bax in the hippocampus

**Figure 3:**
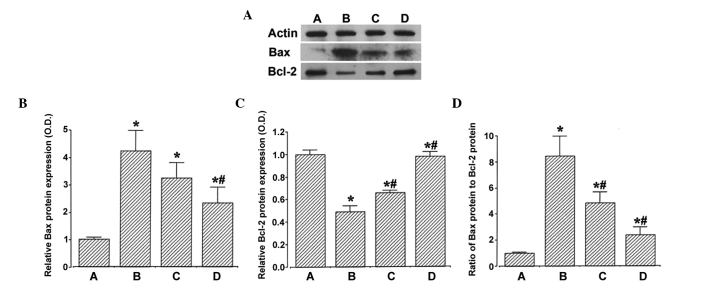
Effects of ulinastatin on the expression levels of Bax and Bcl-2 in the hippocampus. (A) Expression levels of Bax and Bcl-2, detected using western blot analysis. (B) Relative expression of Bax. (C) Relative expression of Bcl-2. (D) Ratio of Bax to Bcl-2. (A) Sham-operation, (B) ischemia-induction, (C) ischemia and 50,000 units ulinastatin, (D) ischemia and 100,000 unitsulinastatin. Data are presented as the mean ± standard error of the mean. *P<0.05, compared with the sham-operation group; ^#^P<0.05, compared with the ischemia-induction group. Blc-2, B cell lymphoma 2; Bax, Bcl-2-associated X protein; OD, optical density.

To verify the effects of ulinastatin on the expression of the apoptosis-associated proteins, the relative expression levels of Bax and Bcl-2 in the hippocampu were determined. When the level of Bax (24 kDa) in the sham group was set at 1.00, the level of Bax was 4.22±0.74 in the ischemia group, 3.24±0.55 in the ischemia and 50,000 units ulinastatin group, and 2.34±0.57 in the ischemia and 100,000 units ulinastatin group. These results revealed that ischemic insult enhanced the expression of Bax in the hippocampus, and that ulinastatin suppressed the ischemia-induced expression of Bax (Fig. 3B).

When the level of Bcl-2 (26–29 kDa) in the sham group was set at 1.00, the level of Bcl-2 was 0.49±0.05 in the ischemia group, 0.66±0.03 in the ischemia and 50,000 units ulinastatin group, and 0.98±0.01 in the ischemia and 100,000 units ulinastatin group. These results indicated that ischemic insult suppressed the expression of Bcl-2 in the hippocampus, and that ulinastatin enhanced the exprssion of Bcl-2 in the ischemic gerbils (Fig. 3C).

### Ratio of Bax to Bcl-2

When the ratio of Bax to Bcl-2 in the sham group was set at 1.00, the ratio of Bax to Bcl-2 was 8.46±1.50 in the ischemia group, 4.85±0.83 in the ischemia and 50,000 units ulinastatin group, and 2.34±0.57 in the ischemia and 100,000 units ulinastatin group. These results demonstrated that ischemic insult enhanced the ratio of Bax to Bcl-2 in the hippocampus, and that ulinastatin suppressed this ischemia-induced increase in the Bax to Bcl-2 ratio (Fig. 3C).

### Effect of ulinastatin on the number of BrdU-positive cells in the hippocampal dentate gyrus

**Figure 4:**
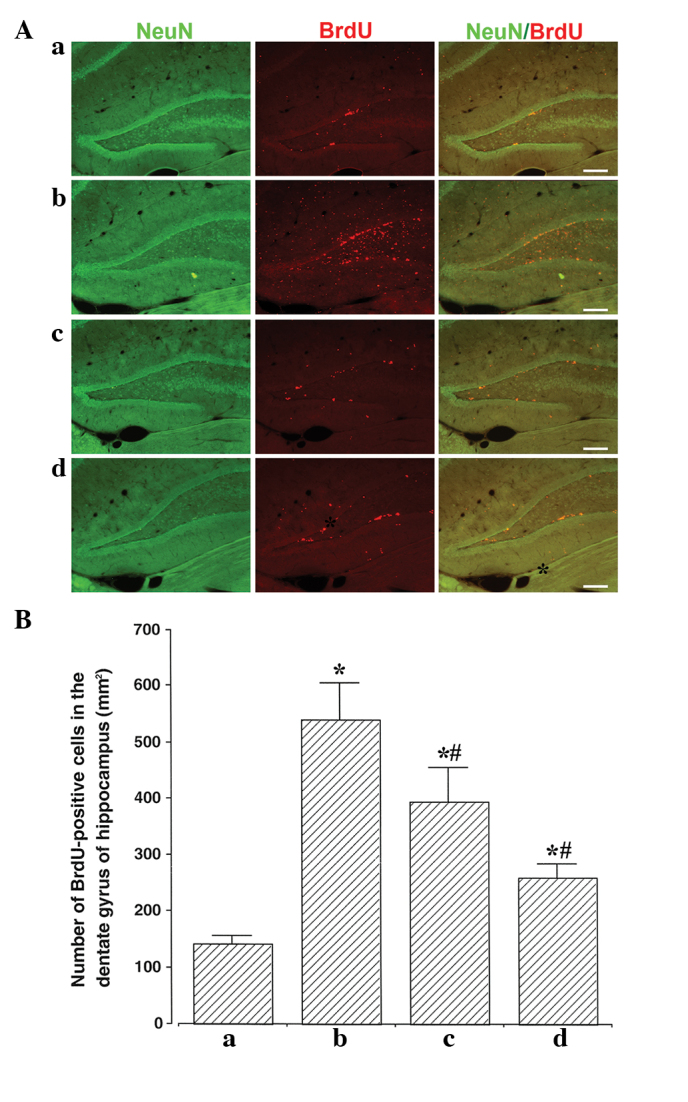
Effects of ulinastatin on cell proliferation in the hippocampal dentate gyrus. (A) Photomicrographs of immunofluorescence for BrdU (red) and neuronal NeuN (green). Scale bars=200 *µm*. (B) Number of BrdU-positive cells. (a) Sham-operation, (b) ischemia-induction, (c) ischemia and 50,000 units ulinastatin, (d) ischemia and 100,000 units ulinastatin. Data are presented as the mean ± standard error of the mean. *P<0.05, compared with the sham-operation group. ^#^P<0.05, compared with the ischemia-induction group. BrdU, 5-Bromo-2′-deoxyuridine; NeuN, nuclear antigen.

Photomicrographs of BrdU-positive cells in the hippocampal dentate gyrus are presented in Fig. 4A. The number of BrdU-positive cells was 141.75±14.53/mm^2^ in the sham group, 540.00±65.56/mm^2^ in the ischemia group, 393.58±61.06/mm^2^ in the ischemia and 50,000 units ulinastatin group, and 260.36±22.84/mm^2^ in the ischemia and 100,000 units ulinastatin group (Fig. 4B). These results revealed that ischemic insult enhanced cell proliferation in the hippocampal dentate gyrus, and that ulinastatin suppressed this ischemia-induced cell proliferation.

## Discussion

The deletion of the CA1 pyramidal neurons is associated with severe impairment of hippocampal-dependent brain functions, including spatial learning ability and short-term memory (4,22,23). Following transient global ischemia in gerbils, short-term memory in the step-down avoidance task is impaired (5). In the present study, latency in the step-down avoidance task was shortened following the induction of transient global ischemia, however, it was improved following treatment with ulinastatin. The results of these experiments indicated that ulinastatin alleviated the short-term memory impairment induced by transient global ischemia in gerbils.

Pyramidal neurons in the hippocampal CA1 region are particularly sensitive to transient global ischemia (24). The upregulation and activation of caspase-3 in the early stages of apoptosis is a hallmark of ischemia (1,6). Increased numbers of TUNEL-positive and caspase-3-positive cells in the hippocampal CA1 region is observed following transient global ischemia in gerbils (5). In the present study, the numbers of TUNEL-positive and caspase-3-positive cells in the hippocampal CA1 region increased following ischemic insult, indicating that cerebral ischemia induced apoptotic cell death in the hippocampal CA1 region. Treatment with ulinastatin suppressed the ischemia-induced increase in DNA fragmentation and expression of caspase-3 in the hippocampal CA1 region, indicating that ulinastatin suppressed cerebral ischemia-induced apoptotic cell death in this region.

The increased expression of Bcl-2 inhibits apoptosis (25), while the overexpression of Bax promotes apoptosis (26). It has been suggested that the balance between Bax and Bcl-2 is imporant in the determination of cell death or survival (27). The induction of intracranial hemorrhage suppresses the expression of Bcl-2 and increases the expression of Bax, resulting in an increase of the Bax to Bcl-2 ratio (10). In the present study, the expression of Bax increased, the expression of Bcl-2 decreased, and the ratio of Bax to Bcl-2 increased following ischemic insult. These results indicated that cerebral ischemia accelerated apoptotic cell death by inhibiting anti-apoptotic molecules and activating pro-apoptotic molecules in the hippocampus. By contrast, treatment with ulinastatin suppressed the expression of Bax and enhanced the expression of Bcl-2 in the hippocampus, resulting in a decrease in the Bax to Bcl-2 ratio. These results indicated that ulinastatin enhanced anti-apoptotic molecules and suppressed pro-apoptotic molecules in the hippocampus.

Increasing neurogenesis in the hippocampal dentate gyrus improves learning ability and memory function (8,11). However, pathological conditions, including brain inflammation, are associated with an increase in neurogenesis without memory enhancement (21,28). In the present study, cell proliferation in the hippocampal dentate gyrus was significantly increased following ischemic attack, indicating that ischemia induced excessive apoptotic neuronal cell death in the hippocampus. Treatment with ulinastatin alleviated the ischemia-induced cell proliferation in the hippocampal dentate gyrus, suggesting that ulinastatin exerted a suppressive effect on the ischemia-induced apoptosis in the hippocampus.

Urinary trypsin inhibitors have been used to treat pancreatitis, septic shock and hemorrhagic shock (29–31). Chen *et al* (32) reported that the protective effects of ulinastatin may be associated with the upregulation of Bcl-2, an inhibitor of cell apoptosis, in a hemorrhagic shock animal model. Sung *et al* (33) reported that ulinastatin exerts analgesic and anti-inflammatory effects by suppressing cyclooxygenase-2 and inducible nitric oxide synthase through the downregulation of nuclear factor κ-B activity in BV2 mouse microglial cells.

In the present study, ulinastatin inhibited ischemia-induced apoptosis in the hippocampus by suppressing apoptosis-associated molecules, thus ameliorating ischemia-induced short-term memory impairment. These results suggested the potential use of ulinastatin as a therapeutic agent for patients with cerebral stroke.
